# Hospital and Institutional News

**Published:** 1913-07-26

**Authors:** 


### HOSPITAL AND INSTITUTIONAL NEWS.

### THE ANNUAL MEETING OF THE BRITISH MEDICAL ASSOCIATION.

The representative meeting of the British Medical Association, held at Brighton at the end of last week and the beginning of this, had a more important agenda to discuss than usual. The constitution heretofore has assigned to the representatives of the divisions the *rôle* of delegates rather than representatives, and this restriction has been found to hamper the work of the Association so much, especially when intricate negotiations such as those connected with the Insurance Act have been in hand, that the representatives have come to the conclusion that it ought to be abolished. The other principal matter in debate was the proposal to assist in forming a trade union, supplementary to or supplanting the Association as it at present exists. Although there are many cogent reasons in favour of such a proceeding, and in particular marked financial advantages, the representatives after a full discussion polled heavily against it; on a card vote there were, for the motion 5,262, against it 11,556. Following the representative meeting, the annual meeting proper began on Tuesday last with a full programme of interesting business. The President's address was delivered on the opening day, that in medicine (Prof. G. R. Murray) on Wednesday, in surgery (Sir Berkeley Moynihan) on Thursday, and the popular lecture (Mr. E. J. Spitta) was down for Friday, July 25.

### TWENTY MILLIONS FOR RESEARCH.

At a dinner of the Anglo-Saxon Club on July 17 the American Ambassador, Dr. W. H. Page, was the guest of honour, and in responding to the toast of the evening he divulged some particulars of what is unquestionably much the biggest scheme on record for medical research and the war against disease. The sum of one hundred million dollars (twenty million sterling) is mentioned as the capital endowment fund of this gigantic enterprise, which is to be world-wide in its scope. There are naturally very few men who could put down so enormous a sum, and there seem good grounds for the belief that the munificent donor is Mr. Rockefeller, certainly one of the few individuals to whom the feat is a possibility. As yet the project has not, it seems, got to a stage at which details can properly be divulged. Comment, therefore, is at present futile; but the most extraordinary views are being aired by people who might be expected to know better. Thus the *Morning Post*, usually one of the best-informed daily papers on scientific matters, asserts that sleeping sickness and hook worm are the same disease, and that the Southern States of America are ravaged by it. This amazing error fairly takes one's breath away, and shows how necessary it is to await full information before attempting to estimate critically the merits of the tremendous research scheme whose conception Dr. Page has disclosed.

### THE OPENING OF NEW PREMISES OF THE NORFOLK AND NORWICH EYE INFIRMARY.

The Duke and Duchess of Norfolk visited the capital town of the county from which their title is taken on July 16 for the purpose of opening the new premises of the Norfolk and Norwich Eye Infirmary. From small beginnings ninety years ago, this institution has progressed steadily in usefulness and reputation, and has for some time outgrown the capacity of the old premises in Pottergate Street. The new site is exactly opposite the Norfolk and Norwich Hospital, to which the infirmary stands in the relation of tenant to landlord. The Duke of Norfolk some few years ago gave a donation of £1,000 to the hospital towards an ophthalmic ward, but with his consent and approval it has now been arranged that all the ophthalmic work of the hospital shall be carried out in the infirmary, which will be an advantage to the patients as well as an economy to both institutions. It is the Duke's £1,000 which has enabled the hospital to buy the site of the infirmary and let it to the latter at a nominal rent. About £1,000 has been received towards the expenses of building; but to complete the equipment and to provide a suitable out-patient department about £2,000 more was required. Towards this the Lord Mayor announced that he himself would give £500, and he appealed to Norwich to provide the balance speedily.

### A MEDICAL POET LAUREATE.

The appointment of Mr. Robert Bridges as Poet Laureate not merely satisfies that tribute of the State to poetry which may be supposed to be the main significance of the office, but is an indirect tribute to medicine, and in particular, to the Royal College of Physicians. Mr. Bridges, who was educated at Eton, and also Corpus Christi College, Oxford, where he graduated M.A. and M.B., left the University for a period of travel, and then studied medicine at St. Bartholomew's Hospital. Born in 1844, he continued his medical career till 1882, when he retired, and his medical appointments in London included the posts of casualty physician at St. Bartholomew's, assistant physician at the Children's Hospital, Great Ormond Street, and physician at the Great Northern Hospital. Before returning to Oxford, near which town he now lives, Mr. Bridges' home was for some time at Yattendon, a Berkshire village, which has already its place on the map of English literature, through the Yattendon Hymnal which he edited. He has always taken a musician's interest in music, and the extremely careful technique of his beautiful verse may have been influenced by his love for a sister art which depends more obviously than poetry upon a science of notation and sound. That this is equally true of poetry, Mr. Bridges has shown by his well-known work on Milton's Prosody, and by his own theories of English accentuation; while his inborn interest in scientific principles may be shown by his remark about his poetic aims at Eton, where, he says, poetry always seemed to him a kind of magic use of words, the secrets of which, like those of other forms of magic, could be learnt by a sufficiently careful student. The scientific frame of mind which we see here applied to poetry has had its own fruits, for instance, the Fellowship of the Royal College of Physicians, which he gained in 1900. Goldsmith, Crabbe, Keats are the three other medical poets best known to the public, unless we add the late Francis Thompson, who was the son of a doctor, and a medical student. Mr. Bridges differs, however, from these in having a medical degree less dubious than Goldsmith's, a poetry less autumnal than Crabbe's, an imagination less sensuous than Keats', an inspiration less exuberant than Francis Thompson's.

### STAFF CHANGES AT GUY'S HOSPITAL.

The announcement last week (p. 476) of staff appointments and changes at Guy's Hospital contained a somewhat curiously worded reference to the "approaching retirement of the senior in age of the physicians." This phraseology was presumably used because it might otherwise have been supposed that the senior physician, Dr. Hale White, was about to retire. In point of fact, it is Dr. Newton Pitt who thus severs his long connection with Guy's, for although junior on the staff to Dr. Hale White, he is older, and thus reaches the retiring age first. Dr. Pitt has been connected with Guy's for over thirty years, and before that was a Fellow of Clare College, Cambridge. He was at one time on the staff at the East London Hospital for Children ("Shadwell"), and also was once physician in charge of the electrical department at Guy's Hospital. Dr. Fawcett has been promoted to fill the vacancy on the full staff, and Dr. G. H. Hunt becomes the new assistant physician in his place. The success of this gentleman has been very rapid, for he first went to Guy's from Oxford in 1907, and was house physician in 1911. Immediately on finishing his house appointments he was appointed medical registrar, and has thus not had long to wait for promotion to the staff. Mr. Chapple, the new assistant gynæcological surgeon, is an Australian who went to Guy's from Cambridge; and Mr. Trethowan, the new orthopædic surgeon, is a Cornishman who took his degree at the University of London.

### THE NEEDS OF QUEEN CHARLOTTE'S HOSPITAL.

At a meeting of the governors of Queen Charlotte's Lying-in Hospital held last week, a resolution was adopted embodying the proposals of the committee of management for a new out-patient department and other additions needed for the more efficient working of the hospital. The Chairman of the committee, in proposing the resolution, pointed out that the number of cases now dealt with is far in excess of those for which accommodation was designed nearly thirty years ago. There is land available for the purpose adjoining the hospital and belonging to it; and the plans for the proposed extensions have been submitted to King Edward's Hospital Fund and passed by that body. There is no need for us to dilate here upon the invaluable work which this celebrated charity carries on; and it will be admitted that the greatest advances in obstetrical science at the present time are taking place along the lines of preliminary care and prophylaxis, for which purposes out-patient accommodation is essential. The proposals adopted by the governors involve an expenditure of about £6,000, towards which about £1,000 has so far been contributed. We commend the scheme to the generous support of the charitable public in general, and to that of fathers and mothers in particular.

### NEW DEPARTMENT AT UNIVERSITY COLLEGE HOSPITAL.

Owing to the generosity of Sir Harry Waechter in granting an annual sum of £300 for five years, University College Hospital will, it is announced, open a special department for the treatment of disease by vaccine therapy. It is thus apparent that University College Hospital is continuing to follow the policy of extended specialisation which has already led to the formation of special departments for diseases of the heart and for tuberculosis. In this new departure they have, of course, the precedent set some years ago by St. Mary's to guide them. It would appear from the signs of the times that medicine is on the threshold of that subdivision into separate specialties which has progressed so far already in surgery. Most of our large hospitals, it is true, have not yet adopted this policy so thoroughly as the authorities at Gower Street seem disposed to; but the London Hospital, for instance, has just started a special department under Dr. Mackenzie for heart diseases, and it is quite probable that in time these pioneer experiments will come to be regarded as absolutely essential parts of any up-to-date and well-organised general hospital.

### INSURANCE CERTIFICATES IN LANCASHIRE.

A compromise has been arrived at in Lancashire between the county Insurance Committee and the panel doctors over the form of certificate to be given concerning insured persons under the Act who are entitled to sickness benefit. The doctors held that they ought not to be asked to specify the exact disease from which the patient suffers, on the ground that this involves a breach of professional confidence and may operate to the prejudice of the patient. The Committee, on the other hand, were told by the approved societies that a mere certificate from a panel doctor without specifying the diagnosis would mean heavy inroads on their funds. Both sides had good cases from their own point of view; and the Insurance Committee have arranged a compromise which will probably work all right, though logically it is ridiculous. By this arrangement the word "illness" will be accepted in lieu of the name of the disease whenever the certifying practitioner considers that a more exact statement will be detrimental to the patient. It is provided, as a consideration for this concession, that in all such cases the doctor will meet in consultation without fee a duly-appointed medical referee.

### A FEVER HOSPITAL LAW SUIT.

The Blyth Urban Council (Northumberland) has successfully defended a lawsuit instituted by the father of a patient who had been under treatment in the fever hospital maintained by that authority. In September last, it came out in evidence, a patient was sent to this hospital certified as suffering from scarlet fever. The medical officer, however, thought she was suffering from diphtheria and not from scarlet fever. About a month later a child was sent to the same hospital suffering from diphtheria, and was put in the same ward as the other patient. This child was treated in the usual way with antitoxin, which was followed some days later by a rash, as is not unusual. After the child was discharged another child in the same family developed scarlet fever, and the first child was then found to be peeling. The theory for the prosecution was that the original patient had scarlet fever, not diphtheria; that by negligence the second patient caught scarlet fever from the first; and that she then passed it on to her sister. The defence was that the first patient did not have scarlet fever; that the second one did not have it either; and that there was no negligence in the conduct of the fever hospital. Between these conflicting contentions the County Court Judge confessed his inability to decide; but as the charges made were certainly not established, he ordered a verdict for the defence.

### TWO MAGNIFICENT BEQUESTS.

The past week has been marked by two splendidly generous bequests to voluntary hospitals. Mr. G. J. Fenwick has bequeathed, free of duty, the sum of £50,000 to the Royal Victoria Infirmary, Newcastle, to be invested as a permanent endowment fund; and also a further legacy of £1,000 for the general purposes of the infirmary. At the time of his death Mr. Fenwick had exchanged the climate of Northumbria for the more equable one of Bournemouth; but he had been a senior partner in a Newcastle bank and also a principal proprietor of a brewery. The other big bequest is that of £25,000, left by Mr. J. S. Fry to the Bristol General Hospital. This gentleman's charitable legacies amount in all to nearly £150,000. Besides the large sum left to the Bristol General Hospital, we note also on the list that the Jubilee Convalescent Home, Bristol, gets £5,000; the Bristol Royal Infirmary, £5,000; the Bristol Hospital for Sick Children and Women, £2,000; the Bristol Voluntary Lock Hospital, £2,000; the Bristol Dispensary, £1,000; and the Bristol Eye Hospital, £2,000. It will thus be seen that institutions for the sick receive rather less than one-third of the total amount left for charitable purposes. Religious bodies and societies receive most of the balance.

### HOSPITALS AT WEIHAIWEI.

Increasing confidence of the Chinese in European methods was shown last year by a very marked rise in the number of patients treated in the hospital at Port Elizabeth, viz., 4,923 out-patients and 110 in-patients, making a total of 5,033, as compared with 3,909. One hundred operations were performed, and of these perhaps the most interesting were ten cases of gunshot wounds received during the fighting in Chinese territory near the frontier of Weihaiwei. There were seven midwifery cases, five of which were difficult instrumental cases. The Chinese are still very conservative in these cases, and very seldom seek assistance, and then only when the patient is *in extremis*. They, as a rule, still prefer the primitive methods of the local beldames, which result in a high rate of mortality among both women and children. The total number of patients treated in the Island Hospital was 1,853. Large numbers of out-patients were treated by Mr. Ma, medical assistant at Wênch'üant'ang, who continues to show great zeal in his work.

### DEATH OF FOUR DOCTORS IN CHINA.

Elsewhere in China, we regret to see, there has been a heavy mortality among medical missionaries from typhus fever. At Sianfu this pestilence has carried off two Fellows of the Royal College of Surgeons—Drs. H. S. Jenkins and C. F. Robertson; at Pekin it has taken Dr. F. Hall; and at Wuhu, Dr. E. H. Hart. The latter had been for eighteen years in charge of the Wuhu General Hospital. Dr. Hall had been for four months Dean of the Faculty of Medicine of the Union Medical College in Pekin, and is believed to have contracted his illness from the patients there. It will thus easily be realised that this quartette of doctors cannot easily be spared or replaced, and that the blow to China, no less than to their relatives and friends, is a heavy one. Highly qualified and experienced as these four doctors were, it is unlikely that they would have neglected any necessary precautions to prevent the contraction of infectious disease; and we can only assume that they received the infection from their patients. The contagion of typhus fever is exceptionally easy to contract in this way, and an epidemic of it almost always claims some victims from amongst nurses or doctors.

### THE BIRMINGHAM SKIN HOSPITAL.

On behalf of the committee of the Birmingham Skin Hospital, Mr. Blackwell, at the last meeting, presented to the ex-Chairman, Mr. William Johnson, an illuminated address expressing the members' high appreciation of Mr. Johnson's valuable services on the committee for sixteen and as chairman for eight years. In making the presentation, Mr. Blackwell spoke of the zeal and devotion with which the many and onerous duties pertaining to the position of chairman had been discharged by his predecessor, who had compelled the admiration of all with whom he had been associated. Dr. F. Vinrace added a tribute of appreciation on behalf of the medical staff of the hospital. In resigning the chairmanship Mr. Johnson does not, we are glad to note, sever his connection with the institution for which he has done so much, but will remain a member of the committee.

### THE SELECT COMMITTEE ON PATENT MEDICINES.

The Report of Sir Henry Norman's Select Committee will, it is hoped, be presented to Parliament before the end of the Session; and it is considered in well-informed quarters that steps may be taken next Session to give effect to its proposals. One of the chief recommendations, it is rumoured, will be the administration of all the regulations concerning proprietary and secret remedies by one body, instead of under several departments as at present. It will probably be agreed also, says the *Times*, to recommend that quack advertisements professing to cure cancer, consumption, and similar deadly maladies shall be prohibited; and that a much more rigorous censorship shall be exercised over medicines advertised to cure feminine ailments. It is thought unlikely that the Committee will advise the publication of the formula of each article on the label or wrapper; but disclosure of such formula to the State authority is expected to be recommended.

### A QUESTION OF AUTHORITY AT BROMSGROVE.

Some degree of resentment appears to have been aroused in the Bromsgrove district by the Chairman of the Worcestershire Insurance Committee. An insured person who was an inmate of the Bromsgrove, Droitwich, and Redditch Hospital made a complaint to the Insurance Committee about his treatment in that institution. No intimation was received by the hospital authorities that an inquiry was to be held, nor were they afforded the chance of being represented. Instead of that, the Chairman of the Worcestershire Committee, Mr. Bund, and a doctor investigated the matter. As a matter of fact they entirely exonerated the hospital, so no harm was done by the fact of the non-representation of the latter. When this was reported to the hospital committee some of the members wanted to take the matter up; but the Chairman persuaded them to let the matter drop, and contented himself with the expression of a hope that in any similar future case notice would be given. Doubtless, private representations to the Worcestershire Insurance Committee will be made on the point, and would certainly be less likely to result in friction than any official expression of the irritation which the Bromsgrove governors feel. But on principle the latter have a strong case against the Insurance Committee.

### THE DEARNESS OF WATER.

London institutions have suffered to a considerable extent through the extra charges made for water by the Metropolitan Water Board, and those in authority have endeavoured to reduce the heavy cost by sinking wells. Difficulties have arisen in some cases, as at Southwark, through manufacturers adopting a similar course, the result being that where two or more people have tapped the same supply, no one has gained entire satisfaction. At Bermondsey the Guardians have expended £2,701 on sinking a well at their infirmary, and have decided to provide a softening and purifying plant. It is extremely improbable that the water will be fit (either at Southwark or Bermondsey) for drinking, but should it be found adaptable for the laundry and for other domestic purposes a great saving will be made in the water accounts of the authorities.

### BEIT FELLOWSHIP FOR AN ARMY SURGEON.

The Trustees of the Beit Memorial Fellowships have, on the advice of the advisory board, initiated a new policy by appointing an Army Medical Corps officer on the active list as a Fellow. The Army Council have approved of the appointment of Captain Marett, R.A.M.C., to this post; and he will undertake research into sand-fly fever in addition to his military duties at Malta. We congratulate both the Army Council and the Beit Trustees on such a sensible arrangement, and one so foreign to War Office methods. The research which Captain Marett will undertake will be into the nature of sand-fly fever, or phlebotomus disease, a minor pestilence which is responsible for much sickness in the Mediterranean Squadron and in Malta and certain parts of India. The *Journal of the Royal Army Medical Corps* has for some years past frequently contained clinical and other reports from officers of the Corps upon this disease; and in fact our Army surgeons can certainly claim to have taken the chief share in establishing its existence. It will, therefore, be all the more appropriate should the credit of isolating the virus and elucidating its life-cycle fall to an R.A.M.C. officer. It is hoped that, incidentally, light will be thrown upon the infecting agents of dengue and yellow fever, which are believed to be closely allied to that of sand-fly fever.

### THE WELSH MEDICAL SCHOOL, CARDIFF.

King Edward VII.'s Hospital, Cardiff, already knows Mr. W. J. Thomas as a munificent benefactor; and indeed there is reason to suppose that his generosity to many forms of charitable enterprise in South Wales is even greater than the public knows of. At any rate, his latest offer is to bear the entire cost of the erection and equipment of the whole of the new physiological buildings required in connection with the Welsh Medical School. There will be no outlay for a site, since that of the old buildings in Newport Road is available; and as it is within five minutes' walk of the hospital, no better situation could well be desired. The designs for this new building are by Colonel E. M. Bruce Vaughan, and the elevation of it published in the *South Wales Daily News* shows that the building will be one which any school might covet. The buildings alone are estimated to cost £10,000 apart from their equipment. It is further hoped that this great gift will secure for the College another grant from the Treasury and from the Cardiff City Council for the medical school, and also stimulate others to support the College.

### JOE MILLER'S GRAVESTONE.

A paragraph in the daily press reminds us that the gravestone of Joe Miller, the celebrated humorist, stands in the hall of King's College Hospital old buildings, closed a few days ago. The explanation of this lies in the fact that the original King's College Hospital (that of 1839, not the 1852 building now closed) was built partly on the churchyard of St. Clement Danes Church. The stone is engraved, "Sacred to the memory of Honest Joe

Miller, who was a tender husband and a sincere friend, a facetious companion and an excellent comedian, who departed this life the 15th day of August, 1738, aged 56 years." It has not been determined, we learn, whether this tombstone is to accompany the hospital to Denmark Hill. Since Joe Miller's only connection with "King's" lies in the building of that hospital over his grave a hundred years after his death, it is difficult to see why his gravestone should be removed to Denmark Hill; and for our own part we shall be exceedingly sorry if such a decision is taken. Probably the authorities of St. Clement Danes could and would provide some suitable resting-place for this monument, which would certainly be a far more desirable destination for it.

### THE FIRST MENTAL HOSPITAL.

The wonderful change in institutions for the care of mental disease, and in the treatment of the patients, is well symbolised in the title mental hospital, which is steadily replacing the word which symbolised equally well the treatment and conditions of the past. It is true that many institutions still keep the old name of asylum, and that where this is kept the visitor will find the same improved atmosphere. The change of name is simply the completion of the process. That is why, now that the change has been made, it seems a pity not to recognise it verbally. Dr. E. S. Pasmore, the medical superintendent of Croydon Mental Hospital, claims for his institution the honour and perspicacity of having been the first asylum to adopt the designation hospital, an honour worth placing on public record, for the first adoption of the new style required both imagination and courage.

### SCHOOL CHILDREN AND COTTAGE HOSPITALS.

The Education Committee of the Acton Urban District Council for some time have been in correspondence with the Acton Cottage Hospital authorities regarding the cost of treating school children for adenoids and enlarged tonsils. The Cottage Hospital required £1 1s. per case, whereas it was stated medical men in Acton would treat such cases for half-a-guinea each. The Secretary of the Education Committee therefore was instructed to communicate with the several medical men in Acton, and from the replies received from five doctors it appeared that they held a meeting before answering, and decided to ask a fee of £1 1s. per case. Subsequently inquiries were made of the Secretary of the West London Hospital, and he replied that, subject to a donation of £5 5s. being made to the hospital, they would supply 40 letters. The Acton Education Committee took this course of action much against their desire, the Council's medical officers having advised that treatment at the local Cottage Hospital was preferable in every way.

### NEW HOSPITAL FOR INFIRMARY.

A special joint committee of the hospital and general purposes sub-committees of the Bath Infirmary have under consideration a scheme for the erection of a new hospital in order to remedy the present overcrowding of the infirmary. In this institution at the present time the infirm are classed with the actual sick needing nursing, and hence a larger staff is required than might be necessary otherwise. Moreover, sick children are treated in the same wards as the older individuals, and advanced with incipient phthisical cases. The scheme is to classify inmates, putting the sick into the proposed new hospital, in which there are to be beds set apart for phthisical cases, and leaving the hopelessly infirm in the existing infirmary. The cost is estimated at £10,000 for 100 beds, which is to be raised by a loan extending over thirty years. It is estimated the sinking fund and interest will cost £578 per annum. It is thought, however, that by the classification of patients £240 annually will be saved, and the nursing expenses subsequent upon that classification will be reduced by some £292, so that the scheme will only involve an extra expenditure of £46 yearly. It is understood that another scheme is under consideration with regard to the remodelling of the heating apparatus and kitchens, which, if adopted, together with the new hospital scheme, would cost £185 per annum, equivalent to one-eighth of a penny rate.

### THE LATE MISS MORTEN.

A striking personality is removed from amongst us by the recent death of Miss Honnor Morten, a pioneer in more than one department of social reform. Institutionally speaking, her best known works were perhaps "Sketches of Hospital Life," "The Nurses' Dictionary," which in other hands has now reached its eighth edition, and her editorial share in "A Complete System of Nursing." But she had many other interests, for she was at one time on the now extinct London School Board, was intensely interested in children, was a prolific lecturer and journalist, and found time to spare for prison reform and woman's suffrage! Nor was she content with talking and writing: as a practical philanthropist she set an example which was as high-minded as her teaching, and her home at Rotherfield was frequently placed at the disposal of helpless and defective children. Indeed her energy in many fields of endeavour was prodigious, and it is astonishing how much she managed to do in the fifty-two years of her life. She was a niece of William Black, the novelist, so literary ability may be said to have run in her blood. Her last book, on Child Nurture, showed evidences of waning powers; and her death was preceded by a long illness.

### MISS EDITH BRADLEY'S NEW APPOINTMENT.

We congratulate the Metropolitan Asylums Board upon the appointment of Miss Edith M. Bradley to the important post of matron of the Eastern Fever Hospital, Homerton. Readers of The Hospital will remember the brave fight and wonderful endurance exhibited by Miss Bradley during the time the Hope Hospital scandals occupied so much space in our columns and those of the local press. She has an excellent knowledge of her work; she is a proved and capable administrator; she has exhibited great tact and ability in bringing to its present state of efficiency the Salford Union (Hope) Hospital, and we congratulate her upon the well-earned promotion which this new appointment has given her. With the valuable and able co-operation of Dr. Austin, the medical superintendent, who was appointed to the Hope Hospital some three years ago, that institution has been brought from a condition which made it almost a byword among Poor-Law institutions to a state of efficiency which entitles it to compare favourably with hospitals of its type throughout the country.

### INSANITARY SHOPS.

In the poorer neighbourhoods of the Metropolis, and in the purlieus of many of our large towns, there are small shops which can only be described as insanitary. All sorts of edibles are sold in them, and milk is often retailed from vessels which are imperfectly protected from germs. There must be, in consequence, a serious risk to health, especially in the more unsavoury neighbourhoods. Of course, there are exceptions, and some of the shopkeepers try their best to keep their shops clean and wholesome, and to protect their edibles from flies and dust. The present condition of things calls, however, for urgent reform, and we are sure that the health of the community would be considerably improved if steps were taken to make the insanitary shops cleaner and more wholesome. This could be done by inspection and a little expenditure of labour, combined with a more methodical and protective arrangement of the edibles.

### A FAMOUS PICTURE FROM THE ARTIST'S POINT OF VIEW.

A famous London surgeon, who must be nameless, was this week visiting a sanatorium, and noticed an engraving of Luke Fildes' famous picture entitled "The Doctor" hanging in the matron's room. "Only yesterday," said he, "I was speaking with the painter of that picture, and I asked for his own interpretation of it. 'Well,' said the artist, 'I wanted to convey to the public something of the splendid spirit which governs the medical profession—the spirit that gives of its best to the rich and poor alike. The picture shows the doctor sitting in a humble cottage, regarding the sick child with concentrated thoughtfulness, thinking and thinking what he could do for it. The child was not a hopeless case, he said, else would the doctor be turning to go, there being nothing to do further for it. I wanted to show that he was sparing no thought because it was poor.'"

### THE DISPENSER'S APPOINTMENT AT BRIGHTON WORKHOUSE.

The Brighton Board of Guardians have adopted the recommendation of a special sub-committee that a dispenser be appointed at a salary of £100 a year, who must be duly registered under the Pharmacy Act, 1868, the officer to be non-resident, to devote his whole time to the duties of the office, and to conform to the regulations put forward by the stock and management committee. The recommendation as originally presented by the sub-committee provided for a female dispenser, but the committee have inserted the words male or female, and thus thrown the appointment open to both sexes. It was pointed out that the proposal involved in this recommendation amounted to a saving of £30 or £40 a year, though the Board would have the whole services of the dispenser.

### THE REVISION OF THE PHARMACOPŒIA.

The address delivered by Mr. J. C. Umney, the president of the British Pharmaceutical Conference at the public meeting held in London this week, dealt with the very important question of the revision of the British Pharmacopœia. The Pharmacopœia is now produced and revised by the General Medical Council, but a large portion of the work of revision is voluntarily performed by the pharmacists forming the Committee of Reference in Pharmacy, of which Mr. Umney is a member. Pharmacists, however, are not officially recognised as having anything to do with the work of revision, although they have for a long time claimed recognition. Mr. Umney announced that in future pharmacists would render no assistance to the General Medical Council unless they were given proper credit for the work they did. He submitted to the Conference meeting a Bill which had been drafted, by which it is proposed to set up a Pharmacopœia Commission, consisting of eight medical men, eight pharmacists, two public analysts, and a barrister; he proposed to submit this Bill to the Privy Council in the hope that it would be adopted by the Government, but, failing that, he proposed that it should be introduced into the House of Commons by the Parliamentary Secretary of the Pharmaceutical Society. The work in connection with the revision of the next Pharmacopœia (which will be published next year) is practically completed, so that there should be ample time to arrange matters between the medical profession and the pharmacists. It is to be hoped that the difficulty will be overcome amicably.

### THIS WEEK'S DRUG MARKET.

The Drug market continues quiet, but the dulness is relieved by the interest which is being taken in the Quinine market. It is believed that at last the agreement between the planters in Java and the quinine manufacturers has been definitely made, and as a consequence an advance in the price of quinine is expected. In any case buyers of quinine could hardly be ill-advised to make their purchases at an early date. The Opium market is also attracting attention, and the view is still held that when the new crop comes on the market prices will be lower. Changes in value have been few. Citric acid still has a dearer tendency. Atropine is tending upwards in value. Cod-liver oil is a quiet market, and prices are practically unchanged.

